# Shikonin sensitizes A549 cells to TRAIL-induced apoptosis through the JNK, STAT3 and AKT pathways

**DOI:** 10.1186/s12860-018-0179-7

**Published:** 2018-12-29

**Authors:** Zhi Lan Guo, Jing Zhe Li, Yan Yan Ma, Dan Qian, Ju Ying Zhong, Meng Meng Jin, Peng Huang, Lu Yang Che, Bing Pan, Yi Wang, Zhen Xiao Sun, Chang Zhen Liu

**Affiliations:** 1 College of Chinese Pharmacy, Beijing University of Chinese Medicine, Beijing, China; 2 Beijing Key Laboratory of Research of Chinese Medicine on Prevention and Treatment for Major Diseases, Experimental Research Center, China Academy of Chinese Medical Sciences, 16 Dong Zhi Men Nei Street, Dong Cheng District, Beijing, China; 3 Department of Geriatric Endocrinology, The General Hospital of Chinese People’s Liberation Army, Beijing, China; 4 Department of Orthopaedics, The General Hospital of Chinese People’s Liberation Army, Beijing, China; 5 Beijing Jiquan Biology Technology Co Ltd., Beijing, China

**Keywords:** Shikonin, TRAIL, Resistant cancers, Sensitization

## Abstract

**Background:**

TRAIL, tumor necrosis factor-related apoptosis-inducing ligand, can selectively kill cancer cells with little or no cytotoxicity toward normal human cells and is regarded as a potential relatively safe antitumor drug. However, some cancer cells are resistant to TRAIL-induced apoptosis. Thus, reagents that potentiate TRAIL-induced cytotoxicity are needed. Herein, we investigated whether shikonin, a natural compound from the root of *Lithospermum erythrorhizon*, can sensitize TRAIL-resistant cells to TRAIL-induced cytotoxicity.

**Results:**

The viability of A549 cells, which were resistant to TRAIL, was significantly decreased after treatment with TRAIL followed by shikonin. The underlying mechanisms by which shikonin sensitizes cells to TRAIL-induced cytotoxicity were also examined. Combined treatment with shikonin and TRAIL activated the caspase and JNK pathways, inhibited the STAT3 and AKT pathways, downregulated the expression of Mcl-1, Bcl-2, Bcl-xL, c-FLIP and XIAP and upregulated the expression of Bid.

**Conclusions:**

In conclusion, the results indicated that shikonin sensitized resistant cancer cells to TRAIL-induced cytotoxicity via the modulation of the JNK, STAT3 and AKT pathways, the downregulation of antiapoptotic proteins and the upregulation of proapoptotic proteins.

## Background

Tumor necrosis factor-related apoptosis-inducing ligand, TRAIL, is a promising antitumor drug because it can induce apoptosis in cancer cells but is minimally cytotoxic to normal cells [1, 2]. However, intrinsic or acquired resistance to TRAIL-induced apoptosis has limited the further utilization of TRAIL in clinical trials [3]. Thus, researchers are studying ways to overcome this resistance, but combination treatments to sensitize resistant cancer cells to TRAIL have been shown effective. Therefore, seeking reagents that can sensitize resistant cancer cells to TRAIL-induced apoptosis is greatly important.

Shikonin, a natural compound isolated from the Chinese herbal plant *Lithospermum erythrorhizon*, has been widely used for thousands of years for the treatment of diverse diseases [4, 5]. Shikonin has recently been demonstrated to have tumoricidal [6, 7] and antiproliferative [8, 9] abilities, to reverse drug resistance to chemotherapy [10–12] and to enhance the cytotoxicity of chemotherapy [11–13]. Moreover, studies have shown that shikonin may be similar to TRAIL in its ability to selectively kill cancer cells while causing minimal cytotoxicity to normal cells [7, 10, 14]. The present study investigated whether shikonin can sensitize TRAIL-resistant cells to TRAIL-induced cytotoxicity and whether combined treatment with shikonin and TRAIL is cytotoxic to normal cells. Furthermore, the underlying mechanism was studied.

The above findings suggest that combined treatment with shikonin and TRAIL may be synergistic in effectively enhancing cytotoxicity and destroying tumors. The mechanisms may operate via processes such as the activation of proapoptotic pathways and the inhibition of prosurvival pathways, with a decrease in the expression of antiapoptotic proteins. The present study is devoted to exploring a reagent that can synergistically promote TRAIL-induced cytotoxicity with minimal toxicity to normal cells; this reagent might be a safe and effective sensitizer useful in TRAIL-based therapy in clinical applications.

## Methods

### Chemicals and reagents

Shikonin (Sigma, USA) was dissolved in dimethyl sulfoxide (DMSO) and stored as a stock solution (50 mM) in aliquots at − 20 °C. Soluble recombinant human TRAIL was purchased from Peprotech. Primary antibodies specific to caspase-3, caspase-8, caspase-9, Mcl-1, Bcl-2, Bcl-xL, Bax, JNK, STAT3, Akt and the phosphorylated forms of JNK, STAT3 and Akt (Ser 473) were purchased from Abcam. Primary antibodies specific to β-actin, c-FLIP, XIAP, Bid, and cleaved caspase-3, − 8, and − 9 were purchased from Cell Signaling Technology.

### Cell culture and treatment

A549 human lung cancer cells (CCL-185, ATCC, USA) were cultured in RPMI-1640, and HEK-293 human embryonic kidney cells (CRL-1573, ATCC, USA) were cultured in Eagle’s minimum essential medium (EMEM). The two cells strains purchase need not ethical approval. All types of media were supplemented with 10% heat-inactivated fetal bovine serum. Cultures were maintained at 37 °C in a humidified atmosphere of 5% CO_2_/95% air.

### Cell viability assay

A549 and HEK-293 cells were seeded into two sets of 96-well plates in triplicate (2 × 10^4^ cells in 100 μl/well). After incubation for 12 h, filter-sterilized shikonin was added to the culture medium in one plate at concentrations of 0, 1, 2, 4, 6 and 8 μM, and 50 ng/ml or 5 ng/ml of soluble TRAIL (sTRAIL) was added to the culture medium in the other plate, with the appropriate controls. After incubation for 12 h, the cell viability in the plate treated with only sTRAIL was assessed by a Cell Counting Kit-8 (CCK8) assay; the shikonin-pretreated plate was then treated with 50 ng/ml or 5 ng/ml sTRAIL for another 12 h, and the cell viability was determined by a CCK8 assay. Furthermore, a long-term cytotoxicity experiment was performed as follows: shikonin (4 μM), alone or supplemented with TRAIL (50 ng/ml), was added to A549 and HEK-293 cells. Cell viability was detected over 96 h at 24 h intervals via a CCK8 assay. Moreover, the pan-caspase inhibitor Z-VAD-FMK (Sigma, USA) was employed for the assessment of the effects of shikonin and TRAIL on A549 cells. After pretreatment with Z-VAD-FMK (50 μM) for 60 min, shikonin and TRAIL were added.

### Annexin V/PI assay

Early indicators of apoptosis were detected by using an annexin V/PI binding kit (Biolegend) and a flow cytometer (Accuri C6, BD Biosciences). In brief, A549 cells were seeded in 6-well plates (4 × 10^5^ cells/well) and treated with shikonin (4 μM) or TRAIL (50 ng/ml) for 12 h. TRAIL-treated cells were collected by trypsinization and then analyzed following the indicated protocol. Flow cytometry was performed within 1 h of cell harvesting. After pretreatment with shikonin (4 μM) for 12 h, A549 cells were then incubated in the presence or absence of 50 ng/ml TRAIL for another 12 h. Then, all the cells were collected and analyzed as above.

### Western blotting

A549 cells were seeded and incubated overnight in a 10 mm culture dish (2.5 × 10^6^/well). Next, shikonin (4 μM) and TRAIL (50 ng/ml) were added to the culture medium. After incubation for 12 h, the TRAIL-treated A549 cells were harvested, and whole cell lysates were prepared with lysis buffer. The shikonin-treated A549 cells were then treated or not treated with 50 ng/ml TRAIL for another 12 h, after which whole cell lysates were prepared.

### Statistical analysis

The data are expressed as the means ± SDs and were analyzed by Student’s t-test using SPSS software to determine the significance of differences between groups. *p* < 0.05 was considered statistically significant.

## Results

### Shikonin enhances TRAIL-induced cytotoxicity

**Fig. 1:**
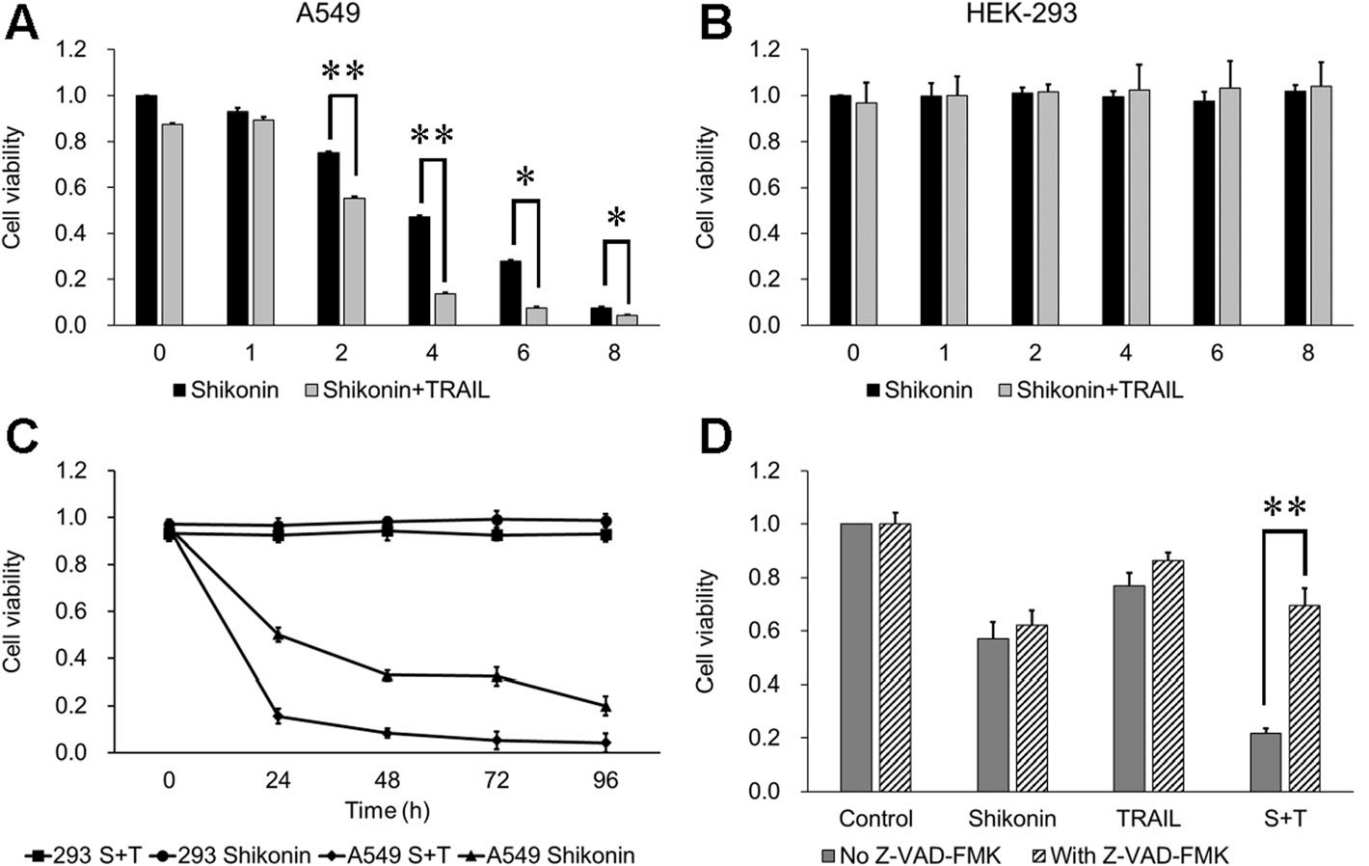
Effect of shikonin on TRAIL-induced cell cytotoxicity. The viability of (**a**) A549 and (**b**) HEK-293 cells after treatment were determined by a CCK8 assay. Cells were pretreated with different concentrations of shikonin for 12 h and then further incubated in the presence or absence of TRAIL (50 ng/ml) for another 12 h. **c** In the long-term cytotoxicity experiment, HEK-293 cell viability was not influenced by shikonin and TRAIL over the 96 h incubation period; however, the viability of A549 cells was significantly inhibited by shikonin, especially with TRAIL cotreatment. **d** These data show the efficacy of shikonin (4 μM) and TRAIL (50 ng/ml) on A549 cells pretreated with Z-VAD-FMK (50 μM). The data are presented as the means ± SDs of at least three independent experiments, *p < 0.05 and **p < 0.01. S + T: Shikonin + TRAIL

After treatment with TRAIL alone, the viability of TRAIL-resistant A549 cells was almost 95%. However, in cells pretreated with shikonin, TRAIL noticeably decreased the viability of A549 cells over time. In addition, a synergistic effect of shikonin and TRAIL on A549 cell killing was observed (Fig. 1a). In addition, the cytotoxicity of shikonin and TRAIL in the normal human renal cell line HEK-293 was assessed. Surprisingly, although shikonin was cytotoxic to the human lung cancer cell line A549, it was almost nontoxic to HEK-293 cells. Moreover, shikonin and TRAIL cotreatment was not cytotoxic to HEK-293 cells (Fig. 1b). The long-term cytotoxicity experiment demonstrated that the concentrations of shikonin and TRAIL were safe in normal cells (Fig. 1c). Z-VAD-FMK, the caspase inhibitor, significantly increased cell viability in the groups cotreated with shikonin and TRAIL (Fig. 1d). Nevertheless, Z-VAD-FMK did not noticeably influence cell viability when shikonin and TRAIL were administered as single reagents.

### Synergistic effect of shikonin and TRAIL on apoptosis induction in A549 cells

**Fig. 2:**
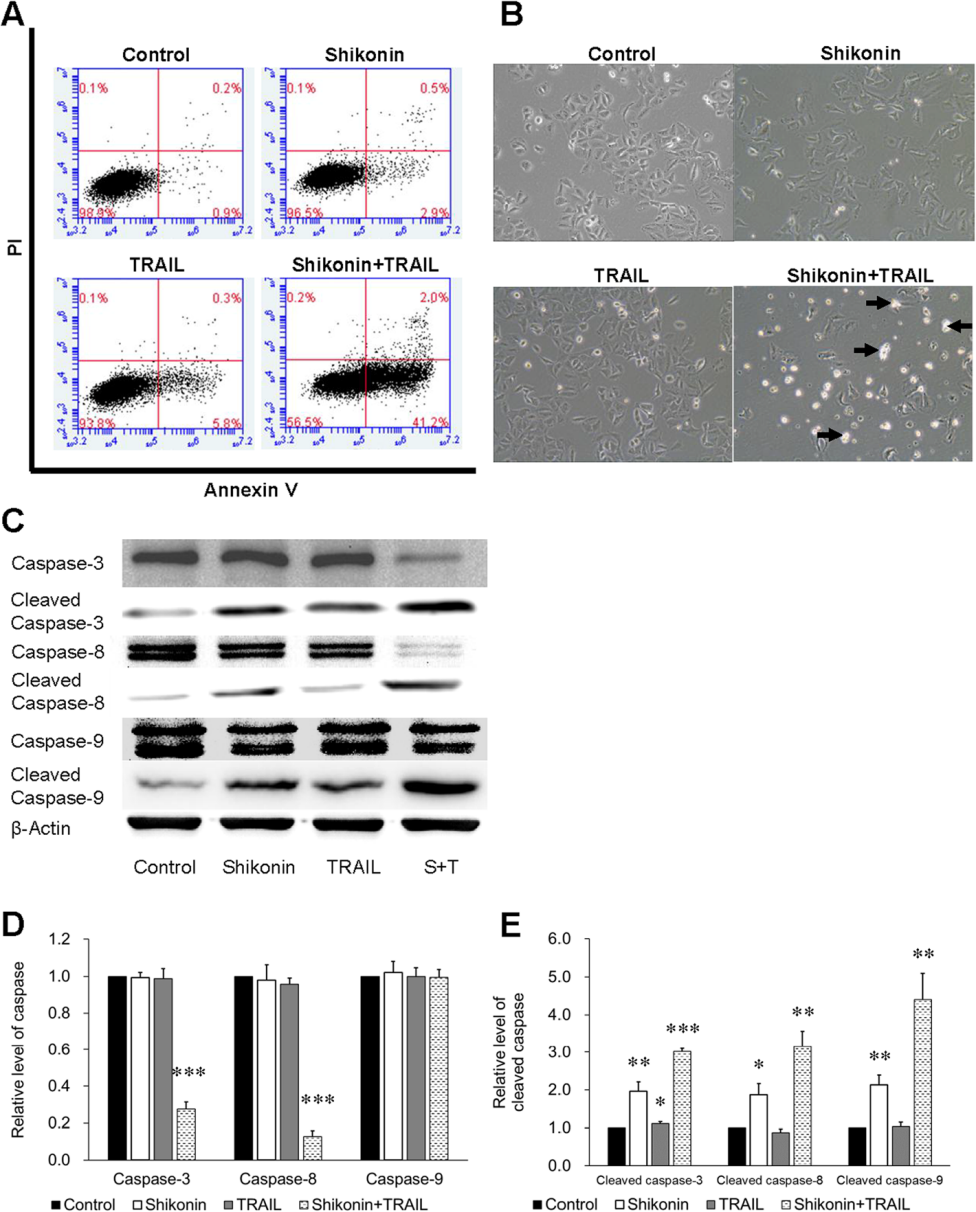
Synergistic effect of shikonin and TRAIL on apoptosis induction in TRAIL-resistant A549 cells. **a** A549 cells were pretreated with shikonin (4 μM) for 12 h and then exposed to TRAIL (50 ng/ml) for another 12 h. Cells were harvested to quantify the induction of apoptosis by annexin V/PI staining and flow cytometry. The data in each plot indicated the percentage of apoptotic cells. **b** Cell morphology was analyzed under an inverted microscope (magnification × 100) after cells were treated as described in (**a**). **c** Whole cell lysates were prepared after cells were treated as described above and were analyzed by Western blotting using antibodies specific to caspase-3, − 8, − 9 and cleaved caspase-3, − 8, − 9. β-Actin was used as the loading control. **d-e** The data are presented as the means ± SDs of at least three independent experiments, and significant differences between the control group and the other groups in each experiment are shown as*p < 0.05, ** *p* < 0.01 and ***p < 0.001. S + T: Shikonin + TRAIL

After the cell viability experiment, a concentration of shikonin (4 μM) was selected for subsequent experiments because this concentration demonstrated the most apparent synergistic effect on TRAIL-induced cytotoxicity in A549 cells. To determine whether shikonin enhanced TRAIL-induced cytotoxicity via apoptosis, apoptosis was quantified by annexin V-PI flow cytometry. After pretreatment with shikonin (4 μM) for 12 h followed by treatment with 50 ng/ml TRAIL for 12 h, the adherent and suspended A549 cells were collected for flow cytometric analysis. The results indicated that treatment with either shikonin (4 μM) or TRAIL (50 ng/ml) alone induced an apoptosis rate of only 3.4% or 6.1%, respectively, while cotreatment with shikonin and TRAIL induced an apoptosis rate of 43.2%; most of the apoptotic cells were in early apoptosis stages (Fig. 2a). Morphological changes in A549 cells after the treatments described above were observed by microscopy; as the arrows in the figure indicated, cellular suspension, shrinkage and blebbing were observed after cotreatment with shikonin and TRAIL, whereas no obvious morphological changes were observed in the groups treated with only shikonin or TRAIL. In addition, the proliferation of A549 cells was inhibited after shikonin treatment. Caspase-3, − 8 and − 9 expression, the hallmarks of cells undergoing apoptosis, was also investigated by Western blotting to further confirm the apoptosis phenomenon. The levels of both pro-caspase-3 and pro-caspase-8 were appreciably decreased in A549 cells after cotreatment with shikonin and TRAIL, while treatment with either shikonin or TRAIL alone had almost no effect on caspase-3 or caspase-8 expression. The Western blotting results showed that cotreatment with shikonin and TRAIL did not activate caspase-9 (Fig. 2c). Taken together, our results indicated that TRAIL-induced apoptosis in A549 cells was augmented after shikonin pretreatment.

### Shikonin potentiates TRAIL-induced apoptosis by inhibiting the expression of antiapoptotic proteins and enhancing the expression of proapoptotic proteins

**Fig. 3:**
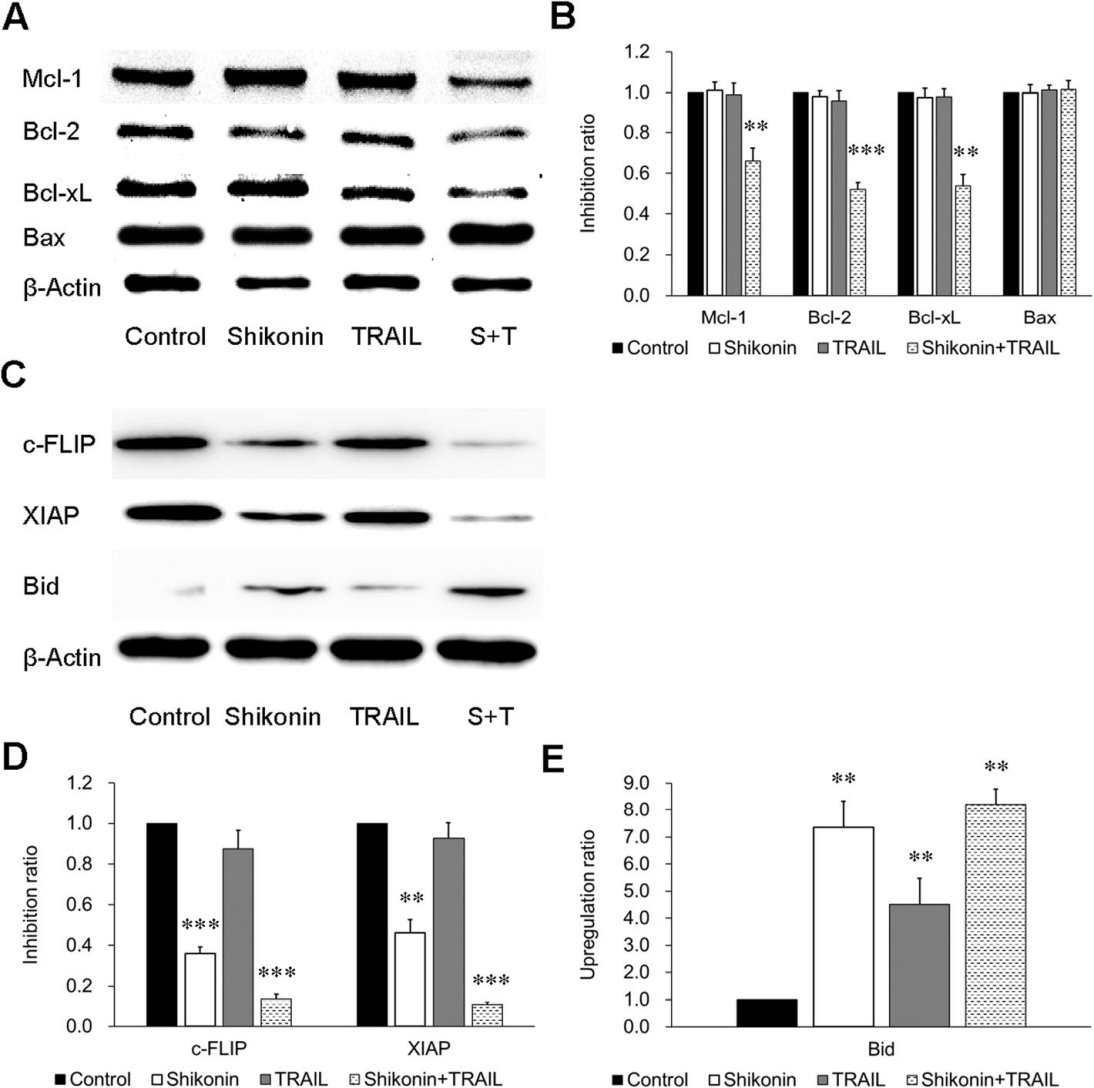
Effect of shikonin and TRAIL on antiapoptotic and proapoptotic protein expression in A549 cells. **a** and **c** A549 cells were pretreated with shikonin (4 μM) for 12 h and were then incubated with TRAIL (50 ng/ml) for another 12 h. Whole cell lysates were prepared and analyzed by Western blotting using the indicated antibodies. β-Actin was used as the loading control. **b, d** and **e** The data are presented as the means ± SDs of at least three independent experiments, and significant differences between the control group and other groups in each experiment are shown as **p < 0.01 and ***p < 0.001. S + T: Shikonin + TRAIL

Next, the mechanisms by which TRAIL-induced apoptosis is enhanced by shikonin were explored. Various anti- or proapoptotic proteins regulate TRAIL-induced apoptosis, so the antiapoptotic proteins Mcl-1, Bcl-2, Bcl-xL, c-FLIP, XIAP and the proapoptotic proteins Bax and Bid were investigated by Western blotting. Cotreatment with shikonin and TRAIL inhibited the expression of the antiapoptotic proteins Mcl-1, Bcl-2, Bcl-xL, c-FLIP and XIAP, while the combination treatment enhanced the expression of the proapoptotic protein Bid but had no effect on Bax expression (Fig. 3). Thus, these results indicated that the downregulation of antiapoptotic proteins and upregulation of proapoptotic proteins were the mechanisms by which shikonin enhanced TRAIL-induced apoptosis.

### JNK, STAT3 and AKT pathways are involved in shikonin-mediated TRAIL sensitization

**Fig. 4:**
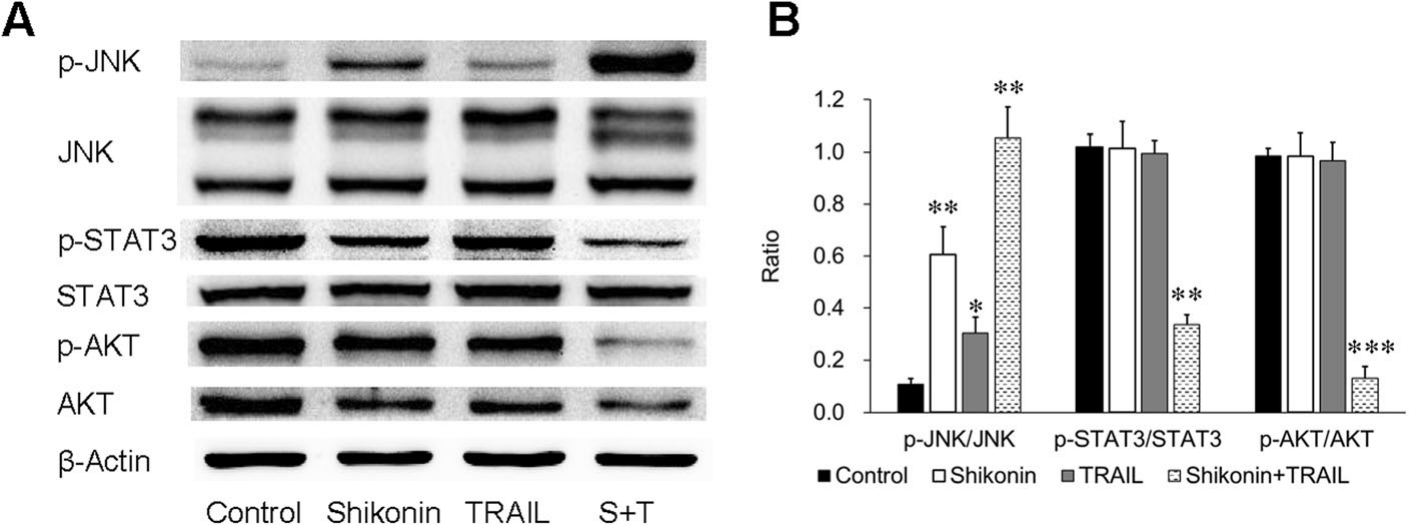
Phosphorylation effect of shikonin and TRAIL on JNK, STAT3 and AKT. **a** Activation of JNK and inhibition of STAT3 and AKT were associated with the induction of apoptosis in A549 cells by shikonin and TRAIL cotreatment as described above. The expression of the total and phosphorylated forms of JNK, STAT3 and AKT were assessed using the corresponding antibodies via Western blotting. **b** The data are presented as the means ± SDs of at least three independent experiments, and significant differences between the control group and the other groups are shown as *p < 0.05, **p < 0.01 *and* ***p < 0.001. S + T: Shikonin + TRAIL

Other signaling pathways, including the JNK, STAT3 and AKT pathways, which were reported to be responsible for resistance to TRAIL, were further investigated. In cells pretreated with shikonin (4 μM) for 12 h followed by treatment with TRAIL (50 ng/ml) for 12 h, Western blotting showed that shikonin upregulated the phosphorylation of JNK (p-JNK); more specifically, the expression of p-JNK was higher after cotreatment than after either treatment alone. On the other hand, shikonin downregulated the phosphorylation of STAT3 and AKT, and this downregulation was more apparent after cotreatment with shikonin and TRAIL (Fig. 4). Therefore, the upregulation of p-JNK and the suppression of p-STAT3 and p-AKT might be the mechanisms by which shikonin sensitizes cells to TRAIL-induced apoptosis.

## Discussion

Cancer has always been one of the most lethal causes of death among human beings. Human beings have to battle cancer for extended periods; surgical excision is the first-line treatment, but many cancers recur after excision. Radiotherapy and chemotherapy are also common treatments for cancers; however, their considerable side effects decrease patients’ quality of life. Thus, relatively safe antitumor drugs that inhibit cancer but have weak or no side effects are urgently needed.

In this study, the role of caspase was the first concern. Cysteinyl aspartate specific protease, or caspase, is a kind of proteolytic enzyme closely related to apoptosis. In apoptosis, the caspase family has two main functions: apoptosis initiation and apoptosis execution [15, 16]. The activated apoptosis-initiating caspase molecule, which is cleaved and activated by an external protein signal, activates the apoptosis-executing caspase molecule and hydrolyzes the target protein, leading to programmed cell death. Therefore, cleaved caspases were investigated, and the results demonstrated that the shikonin + TRAIL drug combination promoted caspase-8 and caspase-3 cleavage. Caspase-8, an apoptosis-initiating molecule, undergoes self-activation through oligomerization and activates downstream caspases, resulting in irreversible apoptosis [17, 18]. Caspase-8 is the key protease in the death receptor pathway. When cells are stimulated by apoptotic factors, TRAIL binds the corresponding death receptor, causing the activation of caspase-8. Then, the downstream caspases can induce a “waterfall” activation process. This action leads to the transfer of the apoptosis signal from the nondependent mitochondrial pathway to the mitochondrial pathway, linking the death receptor pathway with the mitochondrial pathway and amplifying the apoptotic signal [19, 20]. The observation of cleaved caspase-3, − 8, and − 9 was consistent with this theory. Moreover, the pan-caspase inhibitor enhanced cell viability in the shikonin + TRAIL group, proving that shikonin increased the apoptosis-inducing ability of TRAIL via the caspase pathway.

TRAIL, because it can selectively destroy tumor cells but is minimally toxic to normal cells, is regarded as one of the most promising cancer therapeutics [21]. However, drug resistance substantially hinders the efficacy of chemical medicines, and resistance to TRAIL has been reported [22]. Some patients may develop resistance to TRAIL [23, 24], which limits the utilization of TRAIL as a therapeutic reagent in cancer. The mechanism of resistance to TRAIL is not completely understood but may be related to deregulation of the components in the TRAIL-induced apoptotic pathway. Deregulation of TRAIL receptors, including downregulation of the death receptors DR4 and DR5 and overexpression of the decoy receptors DcR1 and DcR2, at the membrane level and deregulation of apoptosis-related proteins, including overexpression of the antiapoptotic proteins Bcl-2, Bcl-xL, and Mcl-1 and downregulation of the proapoptotic proteins Bax, Bid and Bak, at the intracellular level, are responsible for resistance to TRAIL-induced apoptosis [7]. The present results indicated that Bid, a proapoptotic protein, was upregulated, which was believed advantageous to tumor suppression. Bid is activated by Caspase-8 [19]. Our experimental results were consistent with this theory. On the other hand, activation of antiapoptotic pathways, such as the NF-κB, MAPK, PI3K/AKT, and signal transducers and activators of transcription (STAT) pathways, might also confer resistance to TRAIL-induced apoptosis [25]. The role of these pathways is reflected in the experimental results.

Moreover, the caspase, c-FLIP and XIAP proteins play an important regulatory role. c-FLIP is an inhibitor of apoptotic proteins that can inhibit tumor cell apoptosis at high expression levels [26]. The carboxyl terminus of c-FLIP has a structural domain similar to that of caspase-8; thus, c-FLIP competitively binds FADD. Then, c-FLIP regulates multiple apoptotic pathways, including the TRAIL pathway. Therefore, downregulation of c-FLIP was reported to be an effective method for enhancing the sensitivity of tumor cells to TRAIL [27]. Our results indicated that the activity of caspase-3, − 8, and − 9 was increased after c-FLIP expression was suppressed. XIAP, which inhibits apoptotic proteins, mainly mediates protein-protein interactions [28]. Tumor cells can survive and proliferate under adverse conditions, such as nutritional deficiency, hypoxia, DNA damage and chromosomal aberrations. In contrast, these adverse conditions are sufficient to activate apoptosis in normal cells [29]. The process of tumor escape from apoptosis is believed to be the abnormal expression of XIAP in cells. XIAP is an inhibitor of caspase and can selectively bind to caspase-3 and -9 to inhibit cell apoptosis [30]. XIAP can increase the apoptosis threshold, thus playing an important role in mediating tumor amplification and chemotherapeutic drug resistance [31]. In the present study, the expression of XIAP decreased significantly in cells treated with shikonin, especially those cotreated with TRAIL, suggesting that XIAP downregulation may increase tumor sensitization to TRAIL.

Another exciting phenomenon observed was the induction of minimal cytotoxicity in the human embryonic kidney cell line HEK-293 by treatment with shikonin (1–8 μM) either alone or in combination with TRAIL (Fig. 1b). This result is consistent with the report by Gong K et al. [7], in which HEK-293 T and normal human hepatic cells did not undergo cell death after treatment with shikonin (1–8 μM) for 24 h. In addition, several reports indicated that shikonin may be able to selectively kill tumor cells [10, 14] because it can induce intrinsic reactive oxygen species (ROS) upregulation [32, 33]. In addition, compared with normal cells, tumor cells have a higher ROS level and are under oxidative stress due to an imbalanced redox status [34–36], so they are more vulnerable than normal cells to reagents that increase ROS [35]. Thus, the ROS mechanism can be a potential future research direction to better understand the shikonin pathway.

In studying the mechanisms by which shikonin sensitizes cells to TRAIL-induced cytotoxicity, we found that treatment with shikonin followed by TRAIL inhibited the expression of antiapoptotic proteins in the Bcl-2 family: Mcl-1, Bcl-2 and Bcl-xL (Fig. 3). In addition, JNK was activated, and STAT3 and AKT were inhibited. STAT3 is a cytoplasmic transcription factor that plays a critical role in the regulation of genes involved in cell proliferation and survival. Activated STAT3 has been reported to prevent tumor cell apoptosis by regulating associated genes, such as Bcl-2, Bcl-xL and Fas [37, 38]. Furthermore, STAT3 inhibition has been shown to prevent cell proliferation and induce apoptosis in several cancer cell types [39–41]. AKT is a protein kinase involved in multiple cellular processes, including cell survival, proliferation, metabolism, apoptosis and tumorigenesis. AKT suppression has been reported to inhibit proliferation and induce apoptosis in multiple tumor cells [42–44]. As Fig. 2c shows, we observed a phenomenon of growth and proliferation inhibition in the shikonin-treated group; this result might be related to the decreases in activated STAT3 and JNK. Among the above three pathways, the inhibition of the STAT3 and AKT pathways is generally considered beneficial for promoting tumor killing. However, the function of the JNK pathway in tumor killing is controversial [45, 46]. Some findings support the pro-oncogenic function of JNK, while others suggest that JNK is a tumor suppressor [47]. The results of the present study support the latter view.

## Conclusions

The present study demonstrated that shikonin potentiates TRAIL-induced apoptosis by activating JNK and suppressing the activation of STAT3 and AKT, which leads to decreases in antiapoptotic proteins and finally induces extrinsic apoptotic pathways. Importantly, both shikonin and TRAIL selectively kill cancer cells with minimal or no cytotoxicity to normal cells; therefore, combined treatment with shikonin and TRAIL may be an effective and safe strategy for cancer therapy.
